# Antioxidant and angiotensin I-converting enzyme inhibitory activities of Xuanwei ham before and after cooking and *in vitro* simulated gastrointestinal digestion

**DOI:** 10.1098/rsos.180276

**Published:** 2018-07-18

**Authors:** Le Wang, Xiang Li, Yingnan Li, Wenying Liu, Xiaoyun Jia, Xiaoling Qiao, Chao Qu, Xiaoyu Cheng, Shouwei Wang

**Affiliations:** 1 China Meat Research Centre, Beijing 100068, People's Republic of China; 2 Beijing Key Laboratory of Meat Processing Technology, Beijing 100068, People's Republic of China

**Keywords:** Xuanwei ham, cooking, simulated gastrointestinal digestion, antioxidant activity, angiotensin I-converting enzyme inhibitory activity

## Abstract

Xuanwei ham is especially rich in a large amount of peptides and free amino acids under the action of protein degradation. Some of these peptides can potentially exert bioactivities of interest for human health. Traditionally, Xuanwei ham should undergo Chinese household cooking treatments before eating. However, it has not been known how its bioactivity changes after cooking and gastrointestinal digestion. Herein, Xuanwei ham is analysed before and after cooking, as well as gastrointestinal digestion being simulated so as to evaluate and compare its effect on antioxidant and angiotensin I-converting enzyme (ACE) inhibitory activities. The antioxidant activity is analysed using five different methods, and results demonstrate that cooking has some negative effects on antioxidative capacity when determined using different antioxidant methods except for a significant increment in 1,1'-diphenyl-2-picrylhydrazyl radical-scavenging activity, while ACE inhibitory activity increases significantly after cooking compared with control samples. After gastrointestinal digestion of samples, there is a significant increment of the antioxidant and ACE inhibitory activities in comparison with control and cooked samples. Particularly, after gastrointestinal digestion, free thiols content and 2,2'-azino-bis(3-ethylbenzothiazoline-6-sulfonic acid) radical-cation-scavenging activity of Xuanwei ham, respectively, increase about twice and fourfold, while ACE inhibitory activity increases about twice compared to cooked samples, reaching the value of 83.73%. Therefore, through cooking the antioxidant activity and ACE inhibitory activity of Xuanwei ham are not completely lost and a part of them is still maintained, while gastrointestinal digestion produces a significant enhancement in both bioactivities, highlighting a greater potential for a beneficial physiological effect on human health after eating it.

## 1. Introduction

Traditionally, protein in the diet has been considered to be responsible for cellular maintenance, growth and energy. Compared to vegetables and cereals, meat is still one of the more important sources of high-quality protein, essential amino acids and many essential micronutrients in the diet [1]. Generally, dietary proteins have been considered to be potential food for providing bioactive peptides [2], which have been identified from a range of foods, including milk and meat sources such as beef, chicken, pork and fish muscle proteins [3,4]. Bioactive peptides are inactive when they remain within the sequence of the parent protein but exhibit their physiological effect on the body once they are released [5,6]. Peptides in meat can be released by either enzymes, fermentation or ripening during food processing [7]. The specific biological properties of peptides make them an effective functional ingredient that can improve human health, such as by their antioxidant, antihypertensive and anti-inflammatory activity and so on [8]. Antioxidants play an important role in the defence network *in vivo*, which are able to inhibit various disorders and diseases induced by oxidative stress [9,10]. In recent years, some meat-derived antioxidant peptides have been identified through hydrolysis from poultry meat and dry-cured ham [11,12]. In addition, there have been some epidemiological studies suggesting that protein intake or food habit can affect the prevalence of hypertension [13–15]. The most studied mechanism underlying the antihypertensive effects of peptides is inhibition of angiotensin-converting enzyme [16,17]. Angiotensin I-converting enzyme (ACE) is an enzyme of effect in blood pressure regulation through two different reactions of the renin–angiotensin–aldosterone system. ACE inhibitory activity leads to reducing the conversion of angiotensin I into the powerful vasoconstrictor angiotensin II, thus decreasing blood pressure [18]. Therefore, moderate hypertension can be controlled by a nutritional approach and numerous studies have documented antihypertensive and ACE inhibitory effects of different food sources [19–21].

Xuanwei ham is one of the important traditional dry-cured meat products in China with a long history, typical organoleptic, unique flavour and nutritional characteristics [22]. During the ham ripening process, proteolysis is a very important phenomenon, which could be greatly affected by pig genetics as well as conditions such as salt, humidity, temperature and time of ripening. Therefore, the special pig species, climate and terrain in Yunnan province create a superior environment for Xuanwei ham. The action of endogenous enzymes contributes to the generation of large numbers of peptides and free amino acids by degradation of myofibrillar and sarcoplasmic proteins, which have been described to possess special functions for human health [23]. Depending on consumers' preferences, generally Xuanwei ham is not consumed directly. It typically undergoes certain kinds of cooking procedures and thermal treatments before consumption and digestion in the body. Cooking time and temperature greatly influence the physical and chemical properties of protein in Xuanwei ham, such as the extent of protein denaturation, accessibility of enzymes, conformation, functionality, solubility, hydrophobicity and stability, as well as nutritional values [24]. It has been reported that mild short-time heat treatment can increase the antioxidant activity of peptides from pork and beef, but long-term intense heat treatment will cause meat oxidation, resulting in the consumption of antioxidants and reduction of total antioxidant capacity [25]. Simonetti *et al.* [4] have recently reported that the higher thiols content in peptides extracted from the cooked autochthonous pig (75 ± 3°C) may contribute to higher antioxidant stability. Mild thermal treatment (60–80°C) induces protein unfolding, thus enhancing protease susceptibility. Furthermore, a mechanism has been put forward for explaining the increased rate of digestion when the meat is cooked at around 70°C [26]. Around this range of temperature, protein denaturation induces changes in the conformation of proteins, contributing to the bioaccessibility of digestive proteases to their cleaving sites. By contrast, extreme thermal heating leads to irreversible unfolding and aggregation [27]. Therefore, it is of great importance to compare the antioxidant and ACE inhibitory activity of peptides extracted from Xuanwei ham before and after cooking. On the other hand, when cooked meat is in the digestive tract after consumption, its biological activities may be affected by the activity of enzymatic hydrolysis [7]. The released peptides may act in the gastrointestinal tract, while others are absorbed and distributed through the blood stream to target organs and tissues for exerting functions [28,29]. Sangsawad *et al*. [27] recently have reported that simulated gastrointestinal digestion of Korat cross-bred breast meat heated at 70°C for 0.5 h shows higher ACE inhibitory activity compared with other samples. Therefore, simulated digestion of meat proteins with gastrointestinal enzymes is usually applied for investigating the bioaccessibility and availability of bioactive peptides.

Recently, studies have been reported of the antioxidant and ACE inhibitory activities of peptides extracted from Xuanwei ham to a lesser extent and mostly focused on uncooked ham [30]. However, the latter has little impact on human health benefits because Xuanwei ham is not directly consumed considering food safety and palatability. The effect of cooking and digestion of Xuanwei ham is usually disregarded. To the best of the authors’ knowledge, this is the first example for evaluating and comparing the antioxidant and ACE inhibitory activity of peptides extracted from Xuanwei ham before and after cooking and its *in vitro* simulated gastrointestinal digestion. This is of great significance for exploring changes in the biological activity of peptides extracted from Xuanwei ham after cooking and gastrointestinal digestion, and providing a theoretical reference for the nutritional function of Xuanwei ham and the physiological effect on the human body after eating it.

## 2. Material and methods

### 2.1. Chemicals and reagents

Xuanwei hams were provided by Xuanwei Haopin Ham Company (Xuanwei, Yunnan, China). 5,5′-Dithiobis(2-nitrobenzoic acid) (DTNB), ethylenediaminetetraacetic acid (EDTA), 1,1′-diphenyl-2-picrylhydrazyl (DPPH), fluorescein sodium salt, 2,2′-azobis-2-amidinopropane dihydrochloride (AAPH), 6-hydroxy-2,5,7,8-tetramethylchroman-2-carboxylic acid (trolox), ferrozine, ACE (from rabbit lung) and *N*-hippuryl-his-leu hydrate (HHL) were purchased from Sigma Aldrich, Co. (St Louis, MO, USA). The antioxidant capacity assay kit for the 2,2'-azino-bis(3-ethylbenzothiazoline-6-sulfonic acid) (ABTS) method was purchased from Beyotime Biotechnology (Shanghai, China). Pepsin was obtained from Amresco (Solon, OH, USA). Trypsin was obtained from Gibco (Carlsbad, CA, USA). All other chemicals and reagents used were of analytical grade and obtained in China.

### 2.2. Xuanwei ham preparation: control and cooking

#### 2.2.1. Control

**Figure 1.**
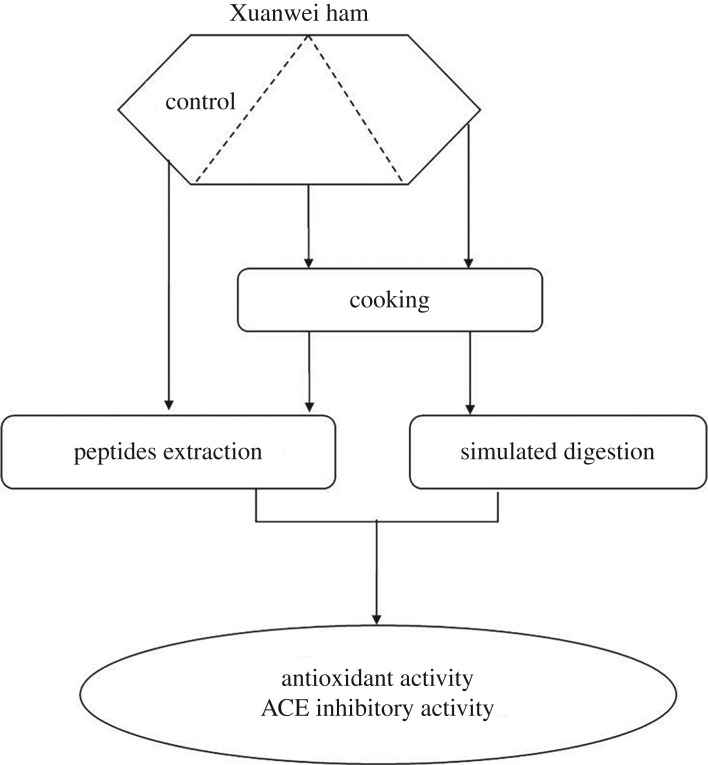
Xuanwei ham sample distribution for the different types of processing and analyses.

Xuanwei hams were prepared according to the traditional procedure involving cooling, salting, washing, drying and fermentation. Xuanwei hams were randomly selected as samples and removed of extramuscular fat and connective tissues. The biceps femoris was fully cut from each ham. Xuanwei ham was used as control, and cooked and digested by simulating gastrointestinal digestion as indicated in figure 1.

#### 2.2.2. Cooking

A part of the Xuanwei ham was taken as control, stored at −20°C until use. The other part of Xuanwei ham was placed in bags and cooked in a water bath at 75°C for 30 min as indicated in figure 1. The internal temperature of cooked Xuanwei ham was measured to be about 70–73°C by thermometer. After cooking, the samples were immediately cooled in an ice batch, and stored at −20°C until use.

### 2.3. Chemical composition analysis of Xuanwei ham

Protein, water and chloride contents of Xuanwei ham were determined according to GB 5009.5-2016, GB 5009.3-2016 and GB 5009.44-2016, respectively.

### 2.4. Peptides extraction of control and cooked Xuanwei ham

The extraction was performed according to Zhu *et al.* [31] with slight modifications. Control and cooked Xuanwei ham (25 g each) were minced and homogenized separately with 100 ml of 0.01 M HCl in a stomacher (Nihonseiki Kaisha, Barcelona, Japan) for 10 min under an ice bath. The homogenate was centrifuged at 12 000*g* for 20 min at 4°C, and then the supernatant was deproteinized by adding three volumes of ethanol and keeping the mixture 20 min at 4°C. Afterwards, the sample was centrifuged at 12 000*g* for 20 min at 4°C again and the supernatant was evaporated in a rotatory evaporator (Four-Ring, China). Finally, the samples were lyophilized. Peptides extracted from control Xuanwei ham and peptides extracted from cooked Xuanwei ham were prepared separately and stored at −20°C until use.

### 2.5. *In vitro* gastrointestinal digestions

*In vitro* gastrointestinal digestion was carried out according to the method of Simonetti *et al*. [4] with minor modification. Cooked Xuanwei ham (25 g) was mixed with 250 ml of deionized water and homogenized for 1 min. Then, the pH was adjusted to 2 with 3 M HCl, and pepsin was added at a 1 : 3000 (enzyme : substrate) ratio. The mixture was incubated at 37°C for 2 h in a shaking water bath. Afterwards, the enzyme was inactivated by adjusting the pH to 7.2 with 1 M NaHCO_3_. Subsequently, trypsin was added at a 1 : 250 (enzyme : substrate) ratio and the mixture was further incubated at 37°C for 3 h in a shaking water bath. Then, enzyme activity was terminated by heating for 10 min at 95°C. The digests were then cooled to room temperature and centrifuged at 5000*g* for 20 min at 4°C; the supernatant was lyophilized and taken as peptides extracted from cooked–digested Xuanwei ham.

### 2.6. Free amino acids analysis

Samples for free amino acids analysis were prepared according to procedures described by Aro *et al*. [32]. Each sample was analysed with an L-8900A amino acid analyser (Hitachi Ltd, Japan).

### 2.7. Free thiol assay

The number of free thiol groups of peptides extracted from control, cooked and cooked–digested Xuanwei ham was determined under the conditions described by Ellman's method [33], with slight modifications. A 250 µl aliquot of each sample solution was mixed with 2.5 ml of 0.1 M sodium phosphate buffer (pH 8.0, containing 1 mM EDTA, reaction buffer); then 50 µl of DTNB solution (4 mg dissolved in 1 ml of reaction buffer) was added. The mixture was shaken evenly and allowed to remain for 30 min at room temperature; the absorbance was measured at 412 nm using a 2800 UV–visible spectrophotometer (Unico, Shanghai, China). The reaction buffer was used instead of the sample as a blank group. The molar extinction coefficient of 14 150 M^−1^ cm^−1^ was applied to estimate the numbers of thiol groups. Thiol content was expressed as nanomoles of free thiol groups per milligram of peptide (nmol SH mg^−1^).

### 2.8. ABTS^•+^ radical-scavenging activity

According to the reported methods, the ABTS assay was carried out using an antioxidant capacity assay kit [34]. ABTS^•+^ solution was generated by a mixing ABTS and potassium persulfate in a 1 : 1 ratio. The mixture was kept for 12–16 h in the dark at room temperature to produce ABTS^•+^ showing green-blue colour, then diluted with phosphate buffer (pH 7.4) to obtain an absorbance of 0.70 ± 0.05 at 734 nm. The ABTS^•+^ radical-scavenging activity of the samples was determined by adding 10 µl of samples into 200 µl of diluted ABTS^•+^ solution in a 96-well plate and then mixing gently. After incubating at room temperature for 6 min, the absorbance of the mixed solution was measured at 734 nm using Synergy™ H4 (BioTek, Vermont, USA). Phosphate-buffered saline was used as control; trolox at different concentrations was used to obtain a standard curve. The ABTS^•+^ radical-scavenging activity was calculated and plotted against the concentration of trolox and the results were expressed as micromoles of TEAC (trolox equivalent antioxidant capacity) per gram of peptide, μmol trolox g^−1^.

### 2.9. 1,1′-Diphenyl-2-picrylhydrazyl radical-scavenging assay

The DPPH radical-scavenging activity of peptides extracted from Xuanwei ham was determined as described by Wang *et al.* [11]. The sample group was obtained by mixing 0.5 ml of each sample solution with 0.5 ml of 0.2 mM DPPH (in 95% ethanol), the control group consisted of aliquots of deionized water mixed 1 : 1 (v/v) with 0.2 mM DPPH (in 95% ethanol) and the blank group consisted of aliquots of samples mixed 1 : 1 (v/v) with 95% ethanol; the mixture was shaken and incubated for 30 min at room temperature in the dark, then the absorbance was measured at 517 nm. The DPPH-scavenging activity was calculated using the following equation: DPPH radical-scavenging activity (%) = 1 − (Abs_sample_ − Abs_blank_)/Abs_control_ × 100%.

### 2.10. Oxygen radical antioxidant capacity assay

The oxygen radical antioxidant capacity (ORAC) assay was determined according to Mawalagedera's method [35]. The reaction was carried out in 75 mM sodium phosphate buffer (pH 7.4). Briefly, 20 µl of each peptide solution and 200 µl of 0.96 µM fluorescein sodium were placed in a well of a 96-well black microplate. The plate was shaken and incubated at 37°C for 10 min. Then, 20 µl of 119 mM AAPH solution was rapidly added and shaken for 30 s before the first reading; the fluorescence at 538 nm was recorded with excitation at 485 nm every 3 min. Trolox at different concentrations was used to obtain a standard curve. The ORAC value was expressed as micromoles of trolox (trolox equivalent) per gram of peptide, μmol trolox g^−1^.

### 2.11. Measurement of Fe^2+^-chelating ability

The Fe^2+^-chelating ability was measured as described by Xing *et al.* [30] with a slight modification. Briefly, 1 ml of the sample solution was mixed with 0.05 ml of 2 mM FeCl_2_ and 0.2 ml of 5 mM ferrozine. The mixture was vortexed and kept at room temperature for 10 min prior to measurement of the absorbance at 562 nm. The chelating activity was calculated as follows: Fe^2+^-chelating ability (%) = (Abs_blank_ − Abs_sample_)/Abs_blank_ × 100%, where Abs_sample_ represented the absorbance of the samples and Abs_blank_ represented the absorbance of the blank in which deionized water replaced the sample solution.

### 2.12. Angiotensin I-converting enzyme inhibitory activity

The assay method of ACE inhibitory activity was modified from that of Hernández-Ledesma *et al.* [36]. A 40 µl aliquot of each peptide solution was added into 100 µl of borate buffer (containing 0.3 M NaCl, 5 mM HHL, pH 8.3) and incubated at 37° C for 3 min. Then, 20 µl ACE solution (0.1 U ml^−1^) was added to each sample solution and the reaction mixture was incubated at 37°C for 30 min. Afterwards, the reaction was terminated by 250 µl of 1 M HCl, and hippuric acid was extracted by the addition of 1.5 ml of ethyl acetate. Following centrifugation (1200*g* for 5 min), 1 ml of supernatant was transferred into a test tube, then heat-evaporated at 95°C to eliminate ethyl acetate, redissolved in 2 ml of deionized water and measured spectrophotometrically at 228 nm. ACE inhibitory activity was calculated as follows: ACE inhibitory activity = [(Abs_a_ − Abs)/(Abs_a_ − Abs_b_)] × 100%, where Abs_a_ is the absorbance when the sample was replaced by borate buffer; Abs_b_ is the absorbance when ACE and the sample were replaced by borate buffer; and Abs was the absorbance of HHL, ACE and the sample.

### 2.13. Statistical analysis

All the tests were repeated in triplicate. Data were evaluated using Excel software. Values were expressed as the mean ± standard deviation (s.d.). Data were statistically analysed using analysis by one-way ANOVA and Duncan's multiple range tests by means of SPSS (standard v. 22.0, SPSS Inc., Chicago, IL, USA). The differences were considered to be significant at *p* < 0.05.

## 3. Results and discussion

### 3.1. Chemical composition analysis of Xuanwei ham

As illustrated in table 1, protein is the most important component of Xuanwei ham. Under the action of endogenous enzymes, complex biochemical changes occurred in muscle protein and a large number of peptides and free amino acids were generated because of protein degradation during the ham ripening process. In addition, the moisture and chloride content of Xuanwei ham were, respectively, 35.40% and 4.77%, which were related to gradual penetration of salt into the muscle tissue. The effective penetration of salt into centre of the muscle tissue of Xuanwei ham was of great significance, which could not only contribute to preventing decay and flavour generation, but also affect the enzyme activity and biochemical reaction in Xuanwei ham; thus the salt content is also closely related to the quality of the ham.

**Table 1.** Chemical composition analysis of Xuanwei ham.

	content %		
	protein	water	chloride
Xuanwei ham	40.40 ± 2.57	35.40 ± 0.82	4.77 ± 0.14

Generally, bioactivities of peptides are closely related to amino acid composition, sequences, hydrophobicity, etc. In this work, we mainly focused on the bioactivity changes of crude peptides and the effect of cooking and *in vitro* simulated gastrointestinal digestion on these parameters, thus mainly using amino acid analysis and hydrophobicity analysis methods as indicated in other reported works in the literature [4,10,37]. Bioactive peptides are usually characterized by short sequences of approximately 2 to 30 amino acids in length and a low molecular weight [1]. As illustrated in table 2, after cooking, the Met, Ile, Leu, Tyr and Phe contents were higher than those of control samples, whereas the content of Asp, Ser, Glu and Lys decreased significantly and the changes of other amino acids were not obvious. Additionally, no significant difference of the content of total free amino acids between peptides extracted from control and cooked Xuanwei ham was observed. It have been reported that hydrophobic amino acid like Val or Leu exposed in peptide sequences could improve the presence of the peptides at the water–lipid interface, thus promoting access for the scavenging of free radicals formed in the lipid [38]. Moreover, amino acid residues such as Met, Glu, Lys and Arg within peptide sequences facilitated the metal-chelating ability of the antioxidant peptides as well as their radical-scavenging potential. In a previous study, the majority of ACE inhibitory peptides have been described to possess Lys or aromatic amino acid residues in each of three positions beside the C-terminal site [39,40]. In this study, hydrophobic amino acids, including Val and Leu, metal-chelating amino acid residues such as Met, Glu, Lys and Arg, and aromatic amino acids like Tyr and Phe occupied more than 60% of the total free amino acids. These amino acids, containing non-polar groups, had a high ability to bind the polyunsaturated fatty acids, which could be conducive to the antioxidant activity of the peptides [41].

**Table 2.** Changes in the free amino acid content of peptides extracted from Xuanwei ham before and after cooking.

free amino acids (mg/100 mg)		
amino acid	control	cooked
Asp	1.25 ± 0.01^a^	1.13 ± 0.03^b^
Thr	0.92 ± 0.01^a^	0.91 ± 0.01^a^
Ser	0.89 ± 0.02^a^	0.85 ± 0.01^b^
Glu	2.29 ± 0.01^a^	2.13 ± 0.04^b^
Gly	0.73 ± 0.02^a^	0.71 ± 0.01^a^
Ala	1.58 ± 0.01^a^	1.60 ± 0.01^a^
Cys	0.04 ± 0.01^a^	0.03 ± 0.01^a^
Val	1.24 ± 0.01^a^	1.26 ± 0.02^a^
Met	0.48 ± 0.03^a^	0.54 ± 0.01^b^
Ile	0.91 ± 0.02^a^	0.97 ± 0.01^b^
Leu	1.79 ± 0.01^a^	1.88 ± 0.02^b^
Tyr	0.55 ± 0.02^a^	0.60 ± 0.01^b^
Phe	0.87 ± 0.01^a^	0.95 ± 0.01^b^
Lys	2.16 ± 0.02^a^	2.06 ± 0.03^b^
His	0.48 ± 0.01^a^	0.49 ± 0.01^a^
Arg	1.21 ± 0.03^a^	1.21 ± 0.01^a^
total	17.37 ± 0.25^a^	17.26 ± 0.22^a^

^a,b^Different superscripts within a row indicate a statistical difference (*p* < 0.05).

### 3.2. The antioxidant activity

Currently, there is no simple universal method for accurate and quantitative assessment of antioxidant capacity. Although there are some methods that could be used to determine the antioxidant capacity, it has been pointed out that many available methods resulted in inconsistent results, and inappropriate application and interpretation of assays because multiple active species and reaction characteristics and mechanisms were involved in oxidative stress [42,43]. Antioxidant activity should not be concluded based on a single antioxidant test model [44]. In addition, considering the complexity and versatility of natural antioxidants *in vivo* and *in vitro*, especially for Xuanwei ham with complicated ingredients, it is more scientific and systematic to comprehensively evaluate antioxidant activity by multiple methods. Generally, most meat-derived antioxidant peptides have demonstrated their properties by a range of *in vitro* assays, which have been widely accepted and reported by an increasing number of studies [37,39,45]. On the basis of the chemical reactions involved, major antioxidant capacity assays were divided into electron transfer (ET) reaction-based methods and hydrogen atom transfer (HAT) reaction [46]. ET-based methods determined the ability of a potential antioxidant to transfer electrons to reduce any compound, including ABTS^•+^ radical-scavenging capacity, DPPH radical-scavenging activity and ferric reducing antioxidant power. HAT-based assays measured the capability of an antioxidant to scavenge free radicals by hydrogen donation to form stable compounds, including the ORAC assay, total radical trapping antioxidant parameter assay and β-carotene bleaching assay. Furthermore, the occurrence of oxidative stress will lead to the decrease of free thiol groups (−SH) and increase of disulfide [47]. This method could reflect the ability of –SH to eliminate free radicals and chelate metal ions. In fact, the loss of −SH in muscle proteins was often used as an indicator of protein oxidation [48].

#### 3.2.1. Free thiol assay

In this study, the antioxidant activity of peptides extracted from control, cooked and cooked–digested Xuanwei ham was calculated by measuring the number of –SH. As presented in table 3, peptides extracted from control Xuanwei ham showed antioxidant activity with a –SH content of 45.09 nmol SH mg^−1^, probably due to the existence of −SH in the amino acid sequence or the exposure of reactive −SH by muscle protein degradation under the action of endogenous enzymes. This was an ideal feature for improving the therapeutic value of Xuanwei ham.

**Table 3.** Free thiols content of peptides extracted from control, cooked and cooked–digested Xuanwei ham (nmol SH mg^−1^).

samples	thiols content (nmol SH mg^−1^)
control	45.09 ± 0.65^a^
cooked	31.24 ± 0.28^b^
cooked + digested	61.67 ± 2.40^c^

^a–c^Different superscripts within a column indicate a statistical difference (*p* < 0.05).

After cooking, the –SH content in peptides extracted from cooked Xuanwei ham decreased significantly (*p* < 0.05), consistent with the results reported by other groups [49]. The –SH content in peptides extracted from cooked Xuanwei ham was 31.24 nmol SH mg^−1^, causing –SH losses of 31%. Many studies have shown that higher temperature range of heat treatment (70–125°C) diminished −SH and increased disulfide content, whereas a gentle heat treatment (less than 50–70°C) of muscle protein could increase −SH, probably due to disruption of disulfide bonds [50]. In previous reports, cooking was shown to induce the formation of reactive oxygen species, and the mercapto amino acids exposed in peptide sequences, because of the high reaction susceptibility of–SH groups, were especially prone to react with these free radicals [51]. The decrease of −SH content has been also reported to be probably caused by the oxidation of accessible free −SH of Cys residues located at the surface of the protein, while the internal Cys residues could be defended from free radical attack even through a prolonged period of heating [52].

After digestion, the –SH content of peptides extracted from cooked–digested Xuanwei ham increased significantly (*p *< 0.05), reaching the value of 61.67 nmol SH mg^−1^, which was increased nearly twice compared to that of cooked samples, indicating a high susceptibility to the action of digestive enzymes. It has been reported that the susceptibility of proteins in cooked meat to the action of proteases depended on the oxidation state of proteins during digestion [52]. In general, gentle oxidation would trigger partial changes of protein structure, leading to an enhancement of its susceptibility to enzymes [53]. In addition, enzymatic hydrolysis promoted the generation of bioactive peptides [54]. Cys-containing peptides like glutathione have also been reported to be released after meat digestion [55]. These peptides were more stable in the gastrointestinal tract than free amino acids because –SH groups were not oxidized, and Cys residues in peptide form were still active even at intestinal pH. Therefore, it can be seen that peptides extracted from control and cooked Xuanwei ham contained a certain amount of –SH content, and the –SH content significantly increased after simulated digestion.

#### 3.2.2. Electron transfer-based antioxidant activity

**Figure 2.**
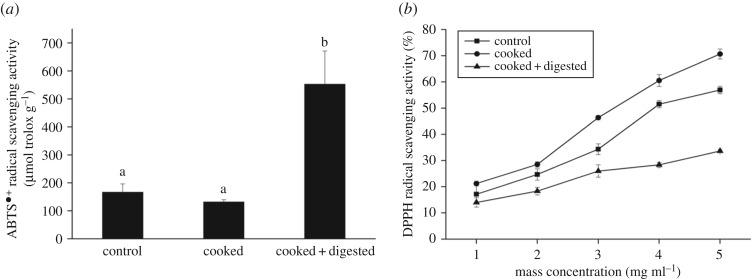
Antioxidant activity of peptides extracted from control, cooked and cooked–digested Xuanwei ham. (*a*) ABTS^•+^ radical-scavenging activity and (*b*) DPPH radical-scavenging activity.

As shown in figure 2*a*, ABTS^•+^ radical-scavenging activity of peptides extracted from control Xuanwei ham was 166.8 µmol trolox g^−1^. After cooking, non-significant differences in ABTS^•+^ radical-scavenging capacity between control and cooked samples were observed. After digestion using gastrointestinal enzymes, the peptides extracted from cooked–digested Xuanwei ham showed a higher ABTS^•+^ radical-scavenging capacity (*p *< 0.05), reaching the value of 552.7 µmol trolox g^−1^, with an increase of about fourfold compared to cooked samples and about threefold compared to control samples, indicating a higher antioxidant activity.

As shown in figure 2*b*, different from the ABTS^•+^ test results, in the DPPH radical-scavenging assay, peptides extracted from Xuanwei ham were shown to have a DPPH radical-scavenging activity which gradually increased with the increase in mass concentration of peptides. The maximum DPPH radical-scavenging activity of control samples in our study was 56.95% at 5 mg ml^−1^. After cooking, DPPH radical-scavenging activity of peptides extracted from cooked Xuanwei ham increased significantly (*p* < 0.05) compared with control samples, reaching the value of 70.68% at 5 mg ml^−1^. After digestion of cooked Xuanwei ham, DPPH radical-scavenging activity was significantly decreased (*p* < 0.05), even significantly lower than the level of control samples.

The results of both DPPH and ABTS^•+^ radical-scavenging activity suggested that the control samples showed antioxidant capacity. This might be related to the peptides containing substances with an electron donor effect, which could react with free radicals to block free radical chain reaction, such as nucleophilic side chains of peptides containing Cys and Met residues. Technological treatments like cooking are particularly important in the development of oxidation and denaturation processes because they might influence the structural characteristics and physico-chemical state of proteins and peptides [52]. After cooking, under oxidation and aggregation of proteins in meat, some hydrophobic amino residues within the peptide sequence could be exposed, leading to an increase of surface hydrophobicity of cooked samples [56]. It has been reported that hydrophobic groups have less ability to attack macromolecules such as proteins in aqueous solution [57]. Therefore, peptides extracted from cooked Xuanwei ham with enhanced hydrophobicity might more easily capture lipid-soluble DPPH radicals compared with control samples. Furthermore, it could be one of the reasons why cooked samples had more interactions with DPPH radicals than water-soluble probes like ABTS^•+^. Consequently, this could explain the higher DPPH radical-scavenging activity obtained by cooked samples compared with control samples. Furthermore, there was slightly decreased ABTS^•+^ radical-scavenging activity but with non-significant differences obtained between control and cooked samples, further confirming the extrapolation of hydrophobicity. When the cooked Xuanwei ham was further digested by simulation with gastrointestinal enzymes, the digest might become more hydrophilic with the accumulation of shorter peptides and amino acids, which could more readily react with hydrophilic ABTS^•+^ radicals rather than lipid-soluble DPPH radicals [58]. This could be a possible reason of why ABTS^•+^ radical-scavenging activity of peptides extracted from cooked–digested Xuanwei ham significantly increased (*p* < 0.05), whereas the result of the DPPH assay significantly decreased after digestion (*p* < 0.05).

#### 3.2.3. Hydrogen atom transfer-based antioxidant activity

**Figure 3.**
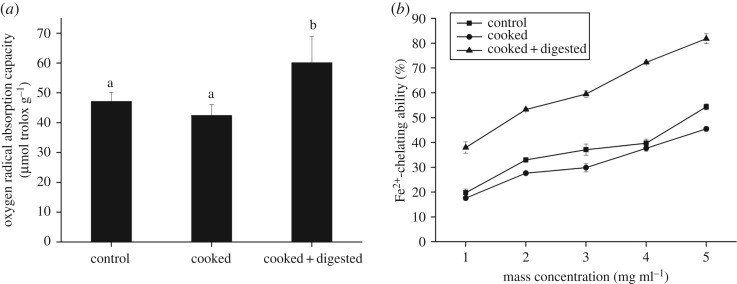
Antioxidant activity of peptides extracted from control, cooked and cooked–digested Xuanwei ham. (*a*) ORAC assay and (*b*) Fe^2+^-chelating ability.

The ORAC assay is a HAT-based method, involving peroxyl radicals as the oxidant and providing useful information on radical chain-breaking capacity [9]. As shown in figure 3*a*, the ORAC value of control samples was approximately 47.13 µmol trolox g^−1^. This is probably because the peptides extracted from Xuanwei ham were rich in hydrogen donors, which possessed the ability of providing hydrogen protons to block the free radical chain reaction, thus achieving the purpose of inhibiting free radicals. No significant differences between control and cooked samples were observed. The results in this study indicated that ORAC values of peptides extracted from cooked–digested Xuanwei ham increased significantly, with an increase of about 42% compared with cooked samples and nearly 30% compared with control samples (*p* < 0.05). Some studies have reported that cooking reduced the antioxidant capacity, whereas simulated digestion could promote the formation of novel antioxidant peptides [12]. The results in this study indicated that the *in vitro* digestion brought an increase in the antioxidant activity of Xuanwei ham digests, and proteins in Xuanwei ham were cleaved to small peptides and free amino acids by gastrointestinal enzymes. A number of studies have proposed that peptides with lower molecular weights presented greater antioxidant activity than their parent native proteins or large polypeptides [58]. Additionally, recent studies have shown that the effect of cooking temperature on the digestion rate of protein was greater than the digestibility, suggesting that the conformational changes caused by denaturation of proteins contributed to the bioaccessibility of digestive protease to cleavage sites [12]. In this study, the trend of changes in the ORAC values of the three samples was consistent with that of previous reports on beef analysed after cooking and simulating digestion [10].

#### 3.2.4. Fe^2+^-chelating ability

Transition metal ions such as iron and copper ions are strong agents to catalyse the generation of free radicals via the Fenton reaction. Therefore, the chelation of metal ions contributes to antioxidation [31]. As shown in figure 3*b*, peptides extracted from Xuanwei ham had Fe^2+^-chelating ability which was gradually increased under a gradient range of concentrations. The control samples exhibited an effective chelating effect on Fe^2+^, reaching 54.37% at the concentration of 5 mg ml^−1^. After cooking, Fe^2+^-chelating ability decreased, whereas after digestion the antioxidant activity considerably increased (*p* < 0.05). When the mass concentration of peptides extracted from cooked–digested Xuanwei ham reached 5 mg ml^−1^, the Fe^2+^-chelating ability achieved the highest chelation rate of 81.84%, which increased nearly twice compared to that of cooked samples and about one and a half times compared to control samples (*p* < 0.05). Some studies showed that basic and acidic amino acids exposed in short peptides may play an important role in Fe^2+^ and Cu^2+^ chelation, such as aspartic acid exposed in short peptides [59]. Therefore, this might be related to peptides extracted from Xuanwei ham containing more basic and acidic amino acids which could chelate Fe^2+^. Heat treatment such as cooking affected the structure of protein in meat, which might change the secondary structure of peptides or reduce the polarity of the peptide, resulting in the decreased Fe^2+^-chelating ability [24]. Following the simulated digestion of cooked Xuanwei ham, the observed high Fe^2+^-chelating ability might be the result of greater exposure of acidic and basic amino acids due to peptide cleavage, because the carboxyl and amino groups in their side chains could bind Fe^2+^. This was probably due to the presence of the large number of acidic amino acids such as Glu and Asp, and basic amino acids including Lys and Arg, and their greater exposure to the external environment after digestion [45].

### 3.3. Angiotensin I-converting enzyme inhibitory activity

In general, bioactive peptides were inactive within the sequence of the parent protein, but could be released in an active form by endogenous enzyme during postmortem, meat processing or proteolysis [1]. As illustrated in table 4, the ACE inhibitory activity of peptides extracted from control Xuanwei ham was 27.65%, indicating that the generation of bioactive peptides might be promoted by the action of protein degradation of muscle after rigor mortis. After cooking, the ACE inhibitory activity increased significantly (*p* < 0.05), which was consistent with the previous reports that the content of bioactive peptides in beef muscle increased after cooking compared to raw samples [60]. The cooking process, accompanied with protein denaturation, triggered the release of peptides due to changes of meat structure, such as myofibril rupture, gel generation of sarcoplasmic proteins or contraction of the connective tissue [61]. Generally, Xuanwei ham was taken after cooking, and thus in order to appraise the effective biological activity of Xuanwei ham, the investigation of the ACE inhibitory activity of changed proteins caused by cooking and their hydrolysates with gastrointestinal enzymes was necessary. After digestion, the ACE inhibitory activity of cooked–digested meat increased significantly, reaching the value of 83.73%, with an increase nearly twice compared to cooked samples and about threefold compared to control samples. This result was in agreement with a report which found that the ACE inhibitory activity of hydrolysate from pork steaks was higher than that of undigested meat [62]. Some research works have shown that drastic proteolysis in gastrointestinal digestion could trigger the generation of many bioactive peptides with low molecular weight [4,62].

**Table 4.** ACE inhibitory activities (%) of peptides extracted from control, cooked and cooked–digested Xuanwei ham.

samples	ACE inhibitory activities (%)
control	27.65 ± 1.74^a^
cooked	45.51 ± 5.15^b^
cooked + digested	83.73 ± 1.92^c^

^a–c^Different superscripts within a column indicate a statistical difference (*p* < 0.05).

## 4. Conclusion

In conclusion, the results showed that Xuanwei ham was a good source of peptides of high nutritional quality with antioxidant activity and ACE inhibitory activity. Peptide bioactivities might be influenced by many factors, such as molecular weight, amino acid composition, structure and hydrophobicity. Results of peptides extracted from cooked Xuanwei ham showed that SH content and Fe^2+^-chelating activity decreased significantly; at the same time respective change in ABTS^•+^ and ORAC radical-scavenging activity was not significant, whereas DPPH radical-scavenging activity and ACE inhibitory activity increasing after cooking compared to control samples, indicating that the peptides extracted from cooked Xuanwei ham still retained certain biological activity although the trend of each evaluation index was not completely consistent after cooking. In addition, after simulating digestion, except that the DPPH radical-scavenging activity reduced significantly, the other antioxidant evaluation methods showed that the antioxidant activity of peptides extracted from cooked–digested Xuanwei ham significantly increased, as well as the ACE inhibitory activity increasing nearly twice compared to cooked samples and about threefold compared to control samples, probably due to the intense generation of small bioactive peptides as a result of the action of gastrointestinal enzymes. Therefore, the simulated gastrointestinal digestion enhanced the nutraceutical quality of Xuanwei ham, which showed higher antioxidant activity and ACE inhibitory activity, highlighting its greater potential for a beneficial physiological effect on human health. This work might not only provide a reference for exploring nutritional quality changes of ham under cooking and digestion, but also expand new perspectives for future investigations to study the physiological effects of consumption of meat.

## Data accessibility

This article has no additional data.
